# Radiation-free automatic planning for cochlear implantation: Comparing cochlear duct lengths between CT and MRI

**DOI:** 10.1016/j.bjorl.2025.101582

**Published:** 2025-04-30

**Authors:** Asma Alahmadi, Fida Almuhawas, Afrah Alshalan, Ibrahim Shami, Eman Hajr, Yassin Abdelsamad, Abdulrahman Alsanosi

**Affiliations:** a Maternity and Children’s Hospital, Makkah Health Cluster, Makkah, Saudi Arabia; b King Abdullah Ear Specialist Center (KAESC), King Saud University Medical City, Riyadh, Saudi Arabia; c Department of Otolaryngology-Head and Neck Surgery, College of Medicine, King Saud University, Riyadh, Saudi Arabia; d Department of Otolaryngology-Head and Neck Surgery, College of Medicine, Jouf University, Skaka, Aljouf, Saudi Arabia; e Department of Otolaryngology-Head and Neck Surgery, Main Hospital, King Fahad Medical City, Riyadh, Saudi Arabia; f Department of Otolaryngology, Imam Mohammad Ibn Saud Islamic University, Saudi Arabia; g Research Department, MED-EL GmbH, Riyadh, Saudi Arabia

**Keywords:** Cochlear implant, Preoperative imaging, Otological planning software, HRCT, MRI, Radiation-free cochlear implantation

## Abstract

**Objective:**

Preoperative imaging is crucial for proper Cochlear Implantation (CI) planning. Currently, no universally accepted guidelines for radiological evaluation existed before CI. While High-Resolution Computed Tomography (HRCT) reveals bony structures but may miss membranous labyrinthine abnormalities, it involves substantial radiation exposure. Conversely, Magnetic Resonance Imaging (MRI) allows visualization of fluid content without radiation exposure. This study aimed to assess the accuracy of automated preoperative cochlear measurements obtained from MRI compared to those extracted manually from HRCT and MRI scans.

**Methods:**

This retrospective study included 55 cochlear implant ears. Preoperative CT and MRI were evaluated using OTOPLAN® to determine the A value, B value, and Cochlear Duct Length (CDL). These measurements were performed using manual (CT and MRI) and automatic (MRI) options. Interrater reliability, concordance, and comparative analyses of the three approaches were conducted.

**Results:**

Our findings indicated that automated MRI measurements were comparable to the manual CT and MRI assessments. No statistically significant differences were detected among the three approaches when determining the A, B, and CDL values. Automated measurements obtained from MRI showed more concordance with CT measurements than those obtained from manual MRI, suggesting that MRI-Auto assesses cochlear parameters better than manual MRI.

**Conclusion:**

Automated cochlear measurements using MRI were comparable to manual assessments using CT and MRI. Moreover, when assessing cochlear metrics, MRI-Auto outperformed the manual MRI workflow. Utilizing MRI-Auto planning for CI surgery can facilitate the process and minimize risks.

**Level of Evidence:**

Level 3.

## Highlights

• Radiation Free CI Planning.
• Utilizing auto-MRI planning for CI surgery facilitates the process and minimize risk.
• Automated cochlear measurement using MRI is comparable to manual CT & MRI assessment.

## Introduction

Cochlear Implants (CIs) are a successful management option for individuals with severe-to-profound sensorineural hearing loss who cannot benefit from hearing aids.^1^, ^2^ Technological advancements have greatly expanded CI candidacy to include other patients, such as adults with eligible degrees of hearing loss and children with congenital anomalies of the inner ear and auditory nerves.^3^, ^4^, ^5^, ^6^ However, many factors affect the postoperative auditory performance of CI recipients, including hearing status before implantation, age at implantation, association with any other disabilities, type and length of the electrode used, and position of placement inside the cochlea.^7^, ^8^ Additionally, human cochleae exhibit inter-individual variations in morphology, coiling characteristics, and size.^9^, ^10^, ^11^ These anatomical variations are associated with considerable differences in the tonotopic organization of the organ of corti along the cochlear duct. Hence, comprehensive preoperative planning for CI, primarily relying on radiological evaluation of the brain and temporal bone, is imperative. This evaluation helps surgeons assess the cochlear anatomy, identify any anatomical abnormalities in the ear, anticipate any potential factors that may contribute to surgical complications, and predict the type and optimal placement of the electrode array.^12^, ^13^

Preoperative imaging is fundamental in determining CI candidacy. It is a common practice to assess the cochlear anatomy, identify congenital anomalies, plan surgery, customize the intraoperative surgical approach, and ensure proper electrode placement.^14^ However, no consensus currently exists regarding the preferred preoperative radiological evaluation modality. Both High-Resolution Computed Tomography (HRCT) and Magnetic Resonance Imaging (MRI) can be used, individually or in combination, depending on institutional guidelines and surgeon preferences.^15^ HRCT provides detailed visualization of cochlear bony structures, including anomalies of the bony labyrinth and cochlear sclerosis,^16^ but may miss up to 10% of soft tissue labyrinth abnormalities such as fibrous obliteration of the cochlea, membranous labyrinth, and endolymphatic sac abnormalities.^17^ In contrast, MRI allows the visualization of the fluid content of the membranous labyrinth, vestibulocochlear nerve hypoplasia or aplasia, cerebellopontine angle, cochlear fibrosis, and inflammation.^14^, ^18^ MRI does not expose patients to radiation, addressing a notable concern associated with HRCT, particularly pediatric patients. CT and MRI can be considered complementary radiological modalities in preoperative CI planning.

OTOPLAN*®* is an otological surgical planning software designed to integrate data from both CT and MRI scans, providing surgeons with crucial information regarding various cochlear parameters.^19^, ^20^, ^21^ Initially, this software required manual labeling of critical cochlear structures (diameter, width, and height), followed by automated computation of Cochlear Duct Length (CDL).^22^ Previous studies have demonstrated the comparable performance of MRI and CT scans using the software's manual option.^20^, ^23^ Nonetheless, one of the common challenges in manual radiological evaluation is the inconsistency in reporting radiological findings owing to inter-rater variability, which could be due to the levels of expertise, training backgrounds, and individual biases.^20^, ^21^, ^22^, ^24^ Hence, a fully Automated algorithm (AUTO) was recently introduced into the software, which has been evaluated only on data integrated from CT scans.^25^

Although MRI can provide all the necessary data for CI planning in addition to being radiation-free compared to CT,^26^ no previous studies have assessed automated measurements obtained from MRI scans. Thus, this study aims to evaluate the precision of automated cochlear measurements obtained from MRI scans compared to those derived from manual workflows using MRI and CT scans.

## Methods

### Study design and setting

This retrospective observational study was conducted at a tertiary hearing implant center. The study protocol was approved by the Institutional Review Board of the College of Medicine (reference number: E-23-7997).

### Participants

The study included patients who (i) Underwent cochlear implant surgery, either unilaterally or bilaterally, between 2019 and 2023; (ii) Had prelingual deafness; (iii) Underwent high-resolution HRCT of the temporal bone and MRI of the inner ear and brain before surgery; and (iv) Had normal cochlear anatomy. Patients who did not fulfill the minimum requirements, those with inner ear anomalies, or those with middle ear disease were excluded.

### Medical imaging

Computed Tomography (CT) and Magnetic Resonance Imaging (MRI) were performed on all patients as part of our institution’s CI candidacy protocol. Temporal bone CT analysis was performed using a 512-slice multidetector-row CT scanner (General Electric Healthcare, Milwaukee, WI, USA). The following scanning parameters were used: axial plane, 0.625-mm slice thickness, 230 mAs, 140 kV, and a rotation time of 1 s with 0.3 mm reconstruction in axial and coronal views. MRI of the Internal Auditory Canal (IAC) and brain was conducted using either a 3.0-T scanner (GE Discovery 750 HD; General Electric Company, Waukesha, WI, USA) or a 1.5-T scanner (GE Sigma 16HDx; General Electric Company). The settings for Fast Imaging Employing Steady-State Acquisition (FIESTA) differed per scanner (3.0-T and 1.5-T, respectively): Frequency matrix: 320 and 320; phase matrix: 256 and 256; number of averages (NEX): 2 and 3; slice thickness: 0.6 and 0.8; overlap: 0.3 and 0.4; Flip Angle (FA): 358 and 658; Field Of View (FOV): 18 and 18; phase FOV: 0.75 and 1; frequency matrix: 320 and 320; phase matrix: 256 and 256; number of averages (NEX), respectively.

### Measurement workflow

The preoperative CT and MRI images were uploaded to a development version of OTOPLAN® V4 (3.0.0) to evaluate several cochlear parameters. Using the software, two independent readers with the same experience level manually labeled the cochlear parameters in CT and MRI images. Furthermore, an automatic software algorithm is employed for automatic image analysis. To run the automatic algorithm, images with voxel size equal to or less than 0.5 × 0.5 × 0.6 are required, with normal inner ear anatomy visible in the picture. Cochlear parameters (A and B values) were evaluated to provide information about cochlear dimensions. The diameter of the cochlea, also known as the A value, is defined as the largest distance from the center of the round window to the lateral wall of the basal turn of the cochlea while traversing the modiolus. The B value, measured perpendicular to the A value line passing through the modiolus, indicated the width of the cochlea.^27^ After defining the cochlear parameters, the software calculated the CDL using the Alexiades method.^16^ applied to the ECA formula^27^

### Statistical analysis

Descriptive statistics were calculated for all measures, such as the mean, standard deviation, and ranges. Intra-Class Correlation coefficients (ICC) were used to evaluate the inter-rater reliability of different cochlear parameters measured using CT or MRI. Using the average from both readers, repeated-measures ANOVA was used to compare the results from CT, manual MRI, and MRI-Auto, followed by multiple Bonferroni-adjusted pairwise *t*-tests. The Concordance Correlation Coefficient (CCC) was estimated to test the degree of concordance between each measuring technique, and correlation analysis of data obtained from MRI-Auto and manual CT- and MRI-based data was performed using Pearson’s correlation coefficients. Normality assumptions were tested using the Shapiro-Wilk test, and a *p-*value ≤ 0.05 was considered statistically significant. Statistical analysis was performed using R software version 4.2.2 “Innocent and Trusting”.

## Results

### Descriptive analysis of assessed cochlear parameters

The study was conducted on 30 cochlear-implanted patients (55 ears); 50.9% (*n* = 28) underwent left-sided implantation, while the remaining 49.1% (*n* = 27) underwent right-sided implantation. Of these patients, 17 were males and 13 were females, averaging 21 years ±20.

Cochlear parameters were assessed using the following approaches: manual features on CT, MRI, and MRI-Auto. To evaluate the reliability of the results, two readings were recorded for all parameters measured manually on both CT and MRI. Concerning the CT scan results, average A values of 8.8 ± 0.6 mm and 8.7 ± 0.6 mm were recorded from the first and second readers, respectively. The first and second readers determined average B values of 6.6 ± 0.4 mm and 6.7 ± 0.4 mm. Therefore, the average CDL calculated from CT data was 34.8 ± 2.1 and 34.7 ± 2.1 mm based on the first and second readers, respectively.

When assessing the cochlear metrics using the manual feature with MRI by two different readers, the A value was measured as an average of 8.9 ± 0.6 mm from the first reader and 8.7 ± 0.6 mm from the second reader. Both readers measured an average B value of 6.7 ± 0.5 mm. Thus, the average CDL calculated from the manual MRI data was 34.9 ± 2.2 mm and 35.0 ± 2.4 mm, as detected by the first and second readers, respectively.

The MRI-Auto measured cochlear parameters recorded an average A value of 8.7 ± 0.6 mm, an average B value of 6.6 ± 0.4 mm (Table 1).

**Table 1:** Descriptive analysis of assessed cochlear parameters.

Measuring techniques		Cochlear parameters		Overall (*n* = 55)
CT measures	First reader	A value (mm)	Mean (SD)	8.8 (0.6)
			Min–Max	7.26–10.32
		B value (mm)	Mean (SD)	6.6 (0.4)
			Min–Max	5.60– 7.59
		CDL (mm)	Mean (SD)	34.8 (2.1)
			Min–Max	29.37–39.83
	Second reader	A value (mm)	Mean (SD)	8.7 (0.6)
			Min–Max	7.42–9.97
		B value (mm)	Mean (SD)	6.7 (0.4)
			Min–Max	5.52–7.84
		CDL (mm)	Mean (SD)	34.7 (2.1)
			Min–Max	30.16–40.50
MRI measures	First reader	A value (mm)	Mean (SD)	8.9 (0.6)
			Min–Max	7.63–10.37
		B value (mm)	Mean (SD)	6.7 (0.5)
			Min–Max	5.74–7.67
		CDL (mm)	Mean (SD)	34.9 (2.2)
			Min–Max	30.22–40.31
	Second reader	A value (mm)	Mean (SD)	8.7 (0.6)
			Min–Max	7.34–10.74
		B value (mm)	Mean (SD)	6.7 (0.5)
			Min–Max	5.72–7.74
		CDL (mm)	Mean (SD)	35.0 (2.4)
			Min–Max	29.73–41.58
MRI-Auto		A value (mm)	Mean (SD)	8.7 (0.6)
			Min–Max	7.43–10.40
		B value (mm)	Mean (SD)	6.6 (0.4)
			Min–Max	5.34–7.59
		CDL (mm)	Mean (SD)	34.6 (2.1)
			Min–Max	29.75–39.98

*Data are represented as mean (standard deviation) and ranges (Min–Max), CDL, Cochlear Duct Length.

### Assessment of inter-observer reliability by comparing CT- and MRI-based data obtained using the manual feature

The Intra-Class Correlation Coefficient (ICC) was calculated between the two readers' manual CT and MRI measurements to assess reproducibility and inter-observer reliability. When evaluating the repeated measures of A and B values and CDL, the ICC demonstrated almost perfect agreement between the two CT readers (A-value: ICC = 0.87, B value: ICC = 0.87, CDL: ICC = 0.92) and between the two MRI readers (A value: ICC = 0.84, B value: ICC = 0.86, CDL: ICC = 0.90). These findings indicate high inter-observer reliability for A values, B values, and CDLs when assessed using CT or MRI (Table 2).

**Table 2:** Assessment of inter-rater reliability for CT and MRI measures performed by two independent readers.

Measuring techniques	Cochlear parameters	ICC coefficients	95% CI
CT	A value	0.87	0.75–0.93
	B value	0.87	0.79–0.92
	CDL	0.92	0.87‒0.95
MRI	A value	0.84	0.72– 0.91
	B value	0.86	0.76–0.91
	CDL	0.9	0.83‒0.94

ICC, Intraclass Correlation Coefficient; CI, Confidence Interval; CDL, Cochlear Duct Length.

### Comparative analysis between MRI-auto and manual CT- and MRI-based data

**Fig. 1:**
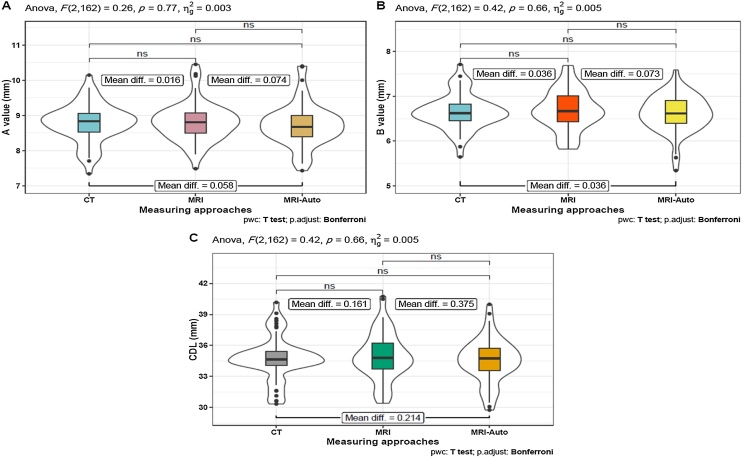
Comparative analysis between MRI-Auto, manual CT-, and MRI-based data. (A) A values, (B) B values, and (C) CDL values. Repeated-measures ANOVA was used to compare the results from three measuring approaches followed by multiple Bonferroni-adjusted pairwise *t*-tests for further detailed analysis. *p-*value ≤ 0.05 was considered statistically significant. *** Indicates < 0.001, * Indicates < 0.05; ns, non-significant.

Our comparative analysis of the three measurement approaches revealed that automatic assessment using MRI-Auto was comparable to manual assessment of CT and MRI . When assessed using repeated-measures ANOVA, no statistically significant differences were detected in the A-value, B-value, and CDL among the three measurement approaches (Table 3 and Fig. 1).

**Table 3:** Comparative analysis between MRI-Auto, manual CT- and MRI-based data.

Cochlear parameters	Group 1	Mean ± SD^a^	Group 2	Mean ± SD^b^	Mean difference	*p-*value^a^	Adj. *p*-value^b^
A value (mm)	CT	8.79 ± 0.55	MRI	8.80 ± 0.56	−0.016	0.773	1.0
	CT	8.79 ± 0.55	MRI-Auto	8.73 ± 0.60	0.058		0.396
	MRI	8.80 ± 0.56	MRI-Auto	8.73 ± 0.60	0.074		0.128
B value (mm)	CT	6.65 ± 0.39	MRI	6.69 ± 0.45	−0.036	0.661	1.0
	CT	6.65 ± 0.39	MRI-Auto	6.62 ± 0.41	0.036		1.0
	MRI	6.69 ± 0.45	MRI-Auto	6.62 ± 0.41	0.073		0.627
CDL (mm)	CT	34.77 ± 2.09	MRI	34.94 ± 2.28	−0.161	0.661	1.0
	CT	34.77 ± 2.09	MRI-Auto	34.56 ± 2.11	0.214		0.666
	MRI	34.94 ± 2.28	MRI-Auto	34.56 ± 2.11	0.375		0.321

CDL, Cochlear Duct Length.

a Family wise *p-*value from ANOVA.

b Adjusted *p*-value from pairwise *t*-test.

### Concordance and correlation analyses of MRI-auto and manual CT- and MRI-based data

Next, we evaluated the degree of precision and accuracy of measurements obtained using MRI-Auto and compared them with those of manual MRI and CT measurements by calculating the Concordance Correlation Coefficients (CCC). A reasonably good degree of concordance was detected between CT, manual MRI, and MRI-Auto measures for A values (MRI: CCC = 0.82, MRI-Auto: CCC = 0.88). A moderate degree of concordance was detected between the two measurement approaches concerning B-values, with concordance coefficients of at least 0.51. The CDL showed satisfactory concordance between the two measurement techniques, with at least 0.69 concordance coefficients. Notably, the concordance between the CT and MRI-Auto measurements was higher than between CT and manual MRI measurements (Table 4).

**Table 4:** Assessment of the degree of concordance between each two measurement approaches.

Cochlear parameters	MRI-Auto vs. CT	MRI-Auto vs. MRI	MRI vs. CT
	CCC (95%CI)	CCC (95%CI)	CCC (95%CI)
A value	0.88 (0.8‒0.92)	0.89 (0.82‒0.93)	0.82 (0.71‒0.89)
B value	0.67 (0.5‒0.79)	0.51 (0.29‒0.68)	0.59 (0.4‒0.74)
CDL	0.81 (0.69‒0.88)	0.69 (0.53‒0.81)	0.7 (0.53‒0.81)

CCC, Concordance Correlation Coefficient, CDL, Cochlear Duct Length.

**Fig. 2:**
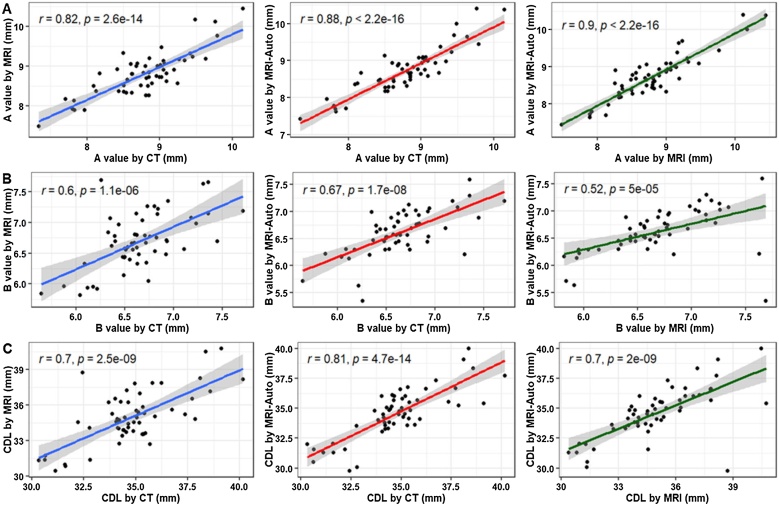
Correlation analysis of the data obtained from MRI-Auto and manual CT- and MRI-based data using Pearson correlation coefficients. (A) A values, (B) B values, and (C) CDL values. *** Indicates < 0.001;** Indicates < 0.05.

Correlation analysis using Pearson’s correlation coefficients revealed statistically significant, very strong positive correlations between CT and manual MRI (*r* = 0.82), CT and MRI-Auto (*r* = 0.88), and manual MRI and MRI-Auto (*r* = 0.9) concerning the A values (Fig. 2A). For the B values, moderate-to-strong statistically significant positive correlations were identified between CT and manual MRI (*r* = 0.6), CT and MRI-Auto (*r* = 0.67), and manual MRI and MRI-Auto (*r* = 0.52) (Fig. 2B). Regarding the CDL, strong to very strong statistically significant positive correlations were detected between CT and manual MRI (*r* = 0.7), CT and MRI-Auto (*r* = 0.81), and manual MRI and MRI-Auto (*r* = 0.7) (Fig. 2C). Taken together, our results from the CCC and correlation analyses indicate that, in comparison to CT, MRI-Auto performs better than manual MRI when evaluating cochlear parameters.

**Fig. 3:**
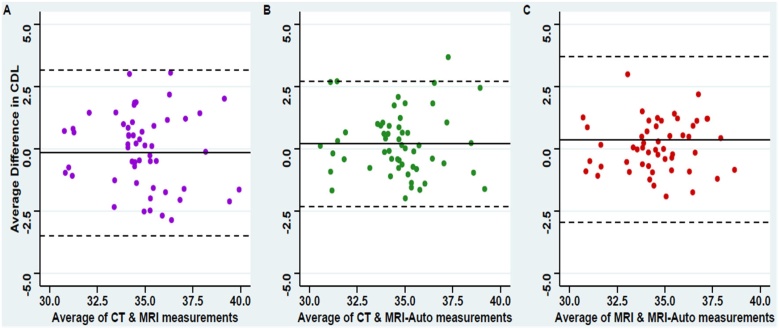
Bland-Altman plot for the agreement between manual CT, MRI, and MRI-Auto estimated cochlear duct length. (A) Showing the agreement between manual CT and MRI, (B) Showing the agreement between manual CT and MRI-Auto, and (C) Showing the agreement between manual MRI and MRI-Auto.

Furthermore, Bland-Altman figures were performed for the agreement between the three measuring approaches regarding the estimated cochlear duct length showed absolute errors around zero between the three approaches with the narrowest 95% Confidence Interval estimate of the mean error between the manual CT and MRI-Auto measures indicating the highest precision of agreement (Fig. 3B) compared to the agreements either between manual CT and MRI (Fig. 3A) or between manual MRI and MRI-Auto (Fig. 3C).

## Discussion

A recent version of OTOPLAN*®* introduces automatic cochlear parameter measurements, which are compatible with both CT and MRI (AUTO).^19^ This study aimed to validate AUTO by comparing the fully automated cochlear measurements obtained from MRI scans (MRI-Auto) with those extracted from the manual option using CT and MRI scans. To our knowledge, this is the first study to validate MRI-Auto as a preoperative cochlear implant planning tool.

Radiological evaluation is crucial in preoperative preparation for CI. Although high-resolution CT is considered the gold standard for inner ear imaging, the radiation dose used in CT raises concerns, mainly when cumulative radiation exposure occurs at a young age.^29^, ^30^ MRI in preoperative CI planning offers several advantages, including a comprehensive anatomical view with excellent inner ear soft tissue contrast without harmful ionizing radiation.^31^ Interestingly, comparable performances of MRI and CT in the preoperative evaluation of CI have been reported.^13^ Together, these findings underline the significance of validating AUTO using MRI as an alternative to CT.

We started this study by recording two different readings for each cochlear parameter using manual features on CT and MRI images of the same patient group and one reading from MRI-Auto. Our cochlear parameter measurements were as follows: the average A value (cochlear diameter) was 8.73 for MRI-Auto and 8.79 mm for both manual options using CT and MRI. These values fall within the range previously reported in a Review Article, which showed that the A values ranged from 7.5 to 10.5 mm, with an average value of 9.0 mm.^28^ Our findings demonstrated that the average B value (cochlear width) for MRI-Auto and manual CT and MR measurements were 6.62 mm and 6.65 mm, respectively. The average CDL calculated by MRI-Auto was 34.56 mm, and those for the manual options using CT and MRI were 34.77 and 34.94, respectively. Our B values and CDL measurements were comparable to those of prior studies, one of which used CT scans to validate AUTO.^25^, ^32^, ^33^ It should be noted that these studies performed cochlear measurements using older versions of the software that only have manual features.

Our comparative analysis demonstrated no significant differences in the measurements of A values, B values, and CDL using the three measurement approaches. More specifically, MRI-Auto assessment was comparable to manual CT and MRI assessment. These results are consistent with previous studies that considered the difference between CT and MRI cochlear metrics to be non-significant.^20^, ^33^, ^34^ However, one study reported significant differences in CDL measurement, with greater values detected by MRI than by flat-panel volume CT measurements.^35^ However, the difference was minor and of questionable clinical significance.

As previously noted in two different studies.^20^, ^25^ Our results revealed excellent inter-observer reliability between the two readings of A values, B values, and CDL performed by two experienced physicians using manual CT (A value: ICC = 0.87, B value: ICC = 0.87, CDL: ICC = 0.92) and MRI (A value: ICC = 0.84, B value: ICC = 0.86, CDL: ICC = 0.90). The high reliability detected between cochlear metrics agrees with other studies, which reported even better inter-rater reliability when assessing the CDL.^22^, ^26^

To gain further insight into the degree of precision and accuracy of the measurements obtained using MRI-Auto, the Concordance Correlation Coefficients (CCC) were calculated compared to measurements derived manually using MRI and CT scans. Our findings revealed that the concordance between the CT and MRI-Auto measurements was higher than between the CT and manual MRI measurements. These findings indicate that MRI-Auto outperforms the manual MRI workflow when evaluating cochlear parameters. The superior performance of MRI-Auto can be justified by its higher accuracy and precision, which offers finer measurements and a more accurate evaluation of cochlear parameters than a manual workflow. Moreover, automation reduces the possibility of subjective interpretation, which may arise from differences in the user experience. Additionally, the Auto feature can save time during the measurement steps and thus facilitate the planning process. Notably, the degree of concordance varied with different cochlear parameters, ranging from good to moderate and satisfactory degrees for A, B, and CDL, respectively.

This study has some limitations, such as the small sample size, and all included MRI scans of normal anatomical structures of the cochleae without any recorded malformations. Therefore, further investigations involving another cohort of patients with cochlear malformations are essential for a comprehensive assessment.

## Conclusion

In conclusion, automatic assessment of cochlear parameters using MRI-Auto using OTOPLAN*®*was comparable to manual CT and MRI assessment. As no clinically significant difference was detected, using MRI-Auto in preoperative planning for CI appears appealing. Moreover, MRI-Auto showed superior performance in evaluating cochlear metrics compared to the manual MRI workflow. This MRI-Auto method for pre-operative planning could be an important step toward radiation-free cochlear implantation.

## Funding

No funding from any organization was received for this work.

## Declaration of competing interest

Yassin Abdelsamad works for MED-EL in scientific roles only, without any commercial or business activities. The authors have no competing interests relevant to the content of this article.
